# How Sensitive Is the Elasticity of Hydroxyapatite-Nanoparticle-Reinforced Chitosan Composite to Changes in Particle Concentration and Crystallization Temperature?

**DOI:** 10.3390/jfb6040986

**Published:** 2015-10-10

**Authors:** Kean Wang, Kin Liao, Kheng Lim Goh

**Affiliations:** 1 Department of Chemical Engineering, The Petroleum Institute, Abu Dhabi, P.O. Box 2533, United Arab Emirates; E-Mail: kwang@pi.ac.ae; 2 Department of Aerospace Engineering, Khalifa University of Science Technology and Research, Abu Dhabi, P.O. Box 127788, United Arab Emirates; E-Mail: kin.liao@kustar.ac.ae; 3 Department of Mechanical Engineering, Khalifa University of Science Technology and Research, Abu Dhabi, P.O. Box 127788, United Arab Emirates; 4 School of Mechanical & Systems Engineering, Newcastle University, Newcastle Upton Tyne, Tyne and Wear NE1 7RU, UK; 5 NU International Singapore, 172A Ang Mo Kio Avenue 8, #05-01, SIT Building@Nanyang Polytechnic, Singapore 567739, Singapore

**Keywords:** particle shape, particle agglomeration, stiffness, strength, toughness

## Abstract

Hydroxyapatite (HA) nanoparticle-reinforced chitosan composites are biocompatible and biodegradable structural materials that are used as biomaterials in tissue engineering. However, in order for these materials to function effectively as intended, e.g., to provide adequate structural support for repairing damaged tissues, it is necessary to analyse and optimise the material processing parameters that affect the relevant mechanical properties. Here we are concerned with the strength, stiffness and toughness of wet-spun HA-reinforced chitosan fibres. Unlike previous studies which have addressed each of these parameters as singly applied treatments, we have carried out an experiment designed using a two-factor analysis of variance to study the main effects of two key material processing parameters, namely HA concentration and crystallization temperature, and their interactions on the respective mechanical properties of the composite fibres. The analysis reveals that significant interaction occurs between the crystallization temperature and HA concentration. Starting at a low HA concentration level, the magnitude of the respective mechanical properties decreases significantly with increasing HA concentration until a critical HA concentration is reached, at around 0.20–0.30 (HA mass fraction), beyond which the magnitude of the mechanical properties increases significantly with HA concentration. The sensitivity of the mechanical properties to crystallization temperature is masked by the interaction between the two parameters—further analysis reveals that the dependence on crystallization temperature is significant in at least some levels of HA concentration. The magnitude of the mechanical properties of the chitosan composite fibre corresponding to 40 °C is higher than that at 100 °C at low HA concentration; the reverse applies at high HA concentration. In conclusion, the elasticity of the HA nanoparticle-reinforced chitosan composite fibre is sensitive to HA concentration and crystallization temperature, and there exists a critical concentration level whereby the magnitude of the mechanical property is a minimum.

## 1. Introduction

Hydroxyapatite is a calcium phosphate salt (Ca_10_(PO_4_)_6_(OH)_2_; HA) which occurs naturally in vertebrate tissues such as bones and teeth [1]. Thus, we can expect it to possess excellent biocompatibility, osteoconduction and osteointegration [2]. Additionally, we note that there are two key characteristics of HA which make it an ideal candidate for blending into and reinforcing the chitosan matrix for applications in tissue engineering [3,4,5]: (1) mechanical stability (when synthesized into the form of nanoparticles with size of 100 nm or less) [6,7] and (2) an affinity to biopolymers [8]. To this end, HA-reinforced chitosan composites in the form of fibres—especially with hierarchical architectures [9] synthesized for consistency with the natural hierarchy of biological tissues [10]—find potential applications in tissue engineering. These findings are well-reported in the literature. For instance, Bhattarai *et al.* [11] demonstrated that chitosan-based nanofibers with an average fiber diameter controllable from a few microns down to 40 nm and that a narrow size distribution could be fabricated by electrospinning. The nanofibers were deposited as a nonwoven mat or as an aligned bundle of controllable size; in either case, the nanofibrous structure demonstrated the capacity to promote the attachment of human osteoblasts and chondrocytes and to maintain the characteristic cell morphology and viability [11]. Zhang *et al*. [12] found that HA/chitosan composite fibre could encourage the growth of cells residing on it; the population of cells increased by 43% (10 days) and 110% (15 days) in scaffolds made from electrospun HA/chitosan fibres. Lai *et al*. [13] demonstrated that the osteogenic differentiation of human mesenchyma stem cells (hMSCs) increased with HA concentration in an HA-reinforced chitosan fibrous scaffold. The study of Lai *et al.* [13] reveals that seeding hMSCs in an HA-reinforced chitosan fibrous scaffold has potential for bone regeneration, and such a scaffold may be used for bone tissue engineering. 

Several methods have been proposed to control the structure of HA nanoparticles during the synthesis process. These methods are known as, to name a few, solid-state reaction [14], sol–gel synthesis [15], pyrolysis of aerosols [16], hydrothermal reaction [17] and microemulsion [18]. Common in some of these methods is the general wet chemical reaction approach that attempts to yield particle precipitation by mixing and then aging at temperatures ranging from room temperature to the boiling point of water [5,17]. HA nanoparticles of the desired structure are obtained via precipitation under a wide range of processing conditions. Nevertheless, the choice of a specific set of values for the processing parameters to synthesize HA with the desired structure for a particular application is not a straightforward process; many studies have reported conflicting results where the optimum levels of the experimental variables are concerned [19,20]. Similar issues also emerged with regard to the synthesis of HA-reinforced chitosan composites. In this study, we are concerned with two key material processing parameters, particle (*i.e.*, HA) concentration and crystallization temperature. Evidence in the literature has shown that these processing parameters affect the stiffness and strength of the HA-reinforced chitosan composite fibres, but most reports involve experiments with either one of the processing parameters as singly applied treatments [5]. Thus, it is not clear how interactions between the two parameters can affect the mechanical properties of the chitosan composite.

Here, our working hypotheses are as follows: (1) HA concentration and crystallization temperature affect the mechanical properties of the HA-reinforced chitosan composite fibre, and (2) the effects of any one factor on the respective mechanical properties are dependent on the other factor. We shall apply the two-factor ANOVA to test these hypotheses, which can also help us gain further insights into the effects of these parameters on HA-reinforced chitosan composites and optimize the values of the processing parameters.

## 2. Results

The results for the mechanical properties (strength, stiffness and toughness) are tabulated in Table 1. From these data, further analysis to investigate the main effects and interaction effects was carried out using the two-factor ANOVA approach. Details of the results are described in the following paragraphs.

**Table 1:** The mechanical properties of the hydroxyapatite (HA)-reinforced chitosan fibres. A total of eight different combinations of crystallization temperature (two levels) and HA concentration (four levels) were explored. The values of the respective mechanical properties are shown as mean ± SEM for informational purposes (SEM: standard error of the mean).

Crystallization Temperature (°C)	HA Concentration (mass fraction)	No. of Specimens	Stiffness (MPa)	Strength (MPa)	Toughness (MPa)
40	0.05	8	2612.6 ± 131.7	34.2 ± 2.2	1.6 ± 0.3
40	0.10–0.15	16	1330.8 ± 87.9	19.7 ± 0.9	1.1 ± 0.2
40	0.20–0.30	16	1213.8 ± 48.3	16.9 ± 0.7	0.7 ± 0.1
40	0.40–0.60	8	1205.3 ± 199.0	20.1 ± 3.1	0.4 ± 0.1
100	0.05	8	1123.5 ± 100.3	19.2 ± 1.3	1.4 ± 0.2
100	0.10–0.15	8	970.1 ± 51.3	16.4 ± 0.9	0.6 ± 0.1
100	0.20–0.30	16	1384.8 ± 90.9	23.9 ± 1.9	0.7 ± 0.1
100	0.40–0.60	16	2899.5 ± 194.6	36.3 ± 2.4	1.5 ± 0.2

**Figure 1:**
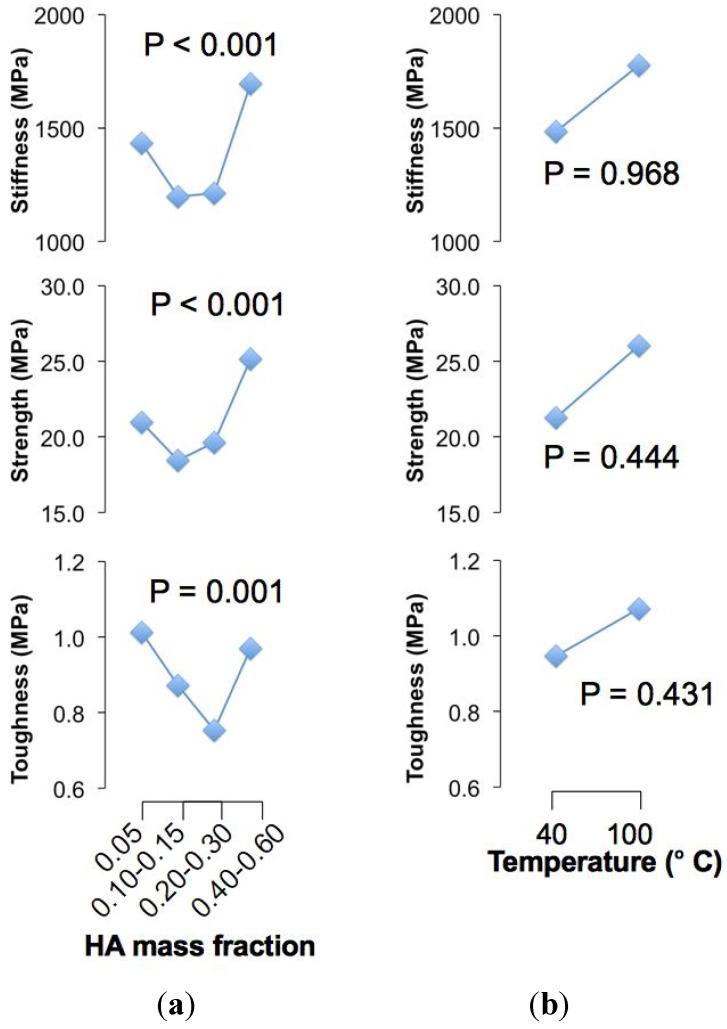
Main effect plots. (**a**) Plot of the mean value of the respective mechanical properties (averaged over the values of the respective specimens for the different crystallization temperatures) *versus* hydroxyapatite (HA) concentration; (**b**) Plot of the mean value of the respective mechanical properties (averaged over the values of the respective specimens for the different HA concentrations) *versus* crystallization temperature.

**Figure 2:**
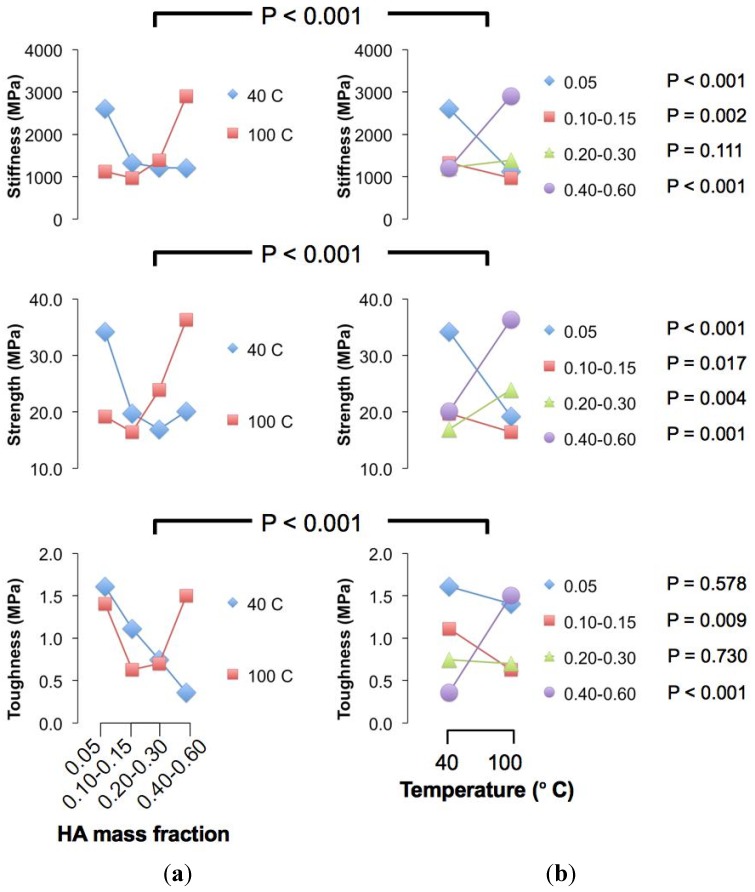
Interaction plots. (**a**) Plot of the mean value of the respective mechanical properties (for each crystallization temperature) *versus* hydroxyapatite (HA) concentration; (**b**) Plot of the mean value of the respective mechanical properties (calculated for each HA concentration) *versus* crystallization temperature. The *P* values for the overall interaction effects test are indicated above each row of graphs. In the right panel, the *P* values to the right of the legends are associated with respective HA concentration.

To begin, we have found significant differences (*P* < 0.05) for the respective stiffness, strength and toughness versus HA concentration but not for the crystallization temperature (*P* > 0.05) (Figure 1). Since there are significant interactions between the two factors (*P* < 0.05) (Figure 2), these suggest that the effects of crystallization temperature on the mechanical properties may be masked by interactions and would require further analysis. 

For the main effects of HA concentration, the mean stiffness is high at low concentration but decreases and remains somewhat unchanged as concentration increases, but beyond 0.30 (HA mass fraction) the mean stiffness increases rapidly (Figure 1). A similar response applies to the strength of the chitosan composite fibre (Figure 1). The mean toughness is high at low concentration but decreases with increase in concentration, attaining a minimum value at the 0.20–0.30 (HA mass fraction) range; thereafter, the mean toughness increases when concentration increases (Figure 1). We conclude that (1) the chitosan composite fibres are stiff, strong and tough at low concentrations, but the stiffness, strength and toughness decrease with increased concentrations; (2) beyond a certain concentration threshold, namely within 0.20–0.30 (HA mass fraction), the values of these mechanical properties increase.

Further analysis using the Student *t*-test (two-sample approach) revealed significant differences in the stiffness of chitosan composite fibre with varying crystallization temperature at low (namely 0.05 (*P* < 0.001), 0.10–0.15 (*P* = 0.002)) and high (namely 0.40–0.60 (*P* < 0.001)) particle concentration levels, but not at intermediate levels (Figure 2). In particular, the stiffness of HA-reinforced chitosan composite fibre increases positively with crystallization temperature at low HA concentrations; the stiffness corresponding to the 40 °C level is higher than that of the 100 °C level, and at very high HA concentration, the reverse applies. 

On the other hand, significant differences (*P* < 0.05) in the fracture strength of chitosan composite fibre with varying crystallization temperature are observed at all HA concentration levels (Figure 2). The trend associate with the magnitudes of the strength with respect to crystallization temperature is similar to the stiffness case. Thus we find that the strength of HA-reinforced chitosan composite fibre increases positively with crystallization temperature at low HA concentration; the strength achieved at the 40 °C level is higher than that at the 100 °C level. However, at very high HA concentrations, the reverse applies.

The toughness of chitosan composite fibre appears less predictable: significant differences (*P* < 0.05) in the toughness with varying crystallization temperature were observed at particle concentration levels of 0.10–0.015 and 0.40–0.60 (HA mass fraction) (Figure 2). In other words, the toughness increases positively with crystallization temperature at around 0.10–0.015 (HA mass fraction) but negatively with crystallization temperature at around 0.40–0.60 (HA mass fraction). However, no significant differences were observed at HA concentration levels of 0.05 (*P* = 0.578) and 0.20–0.30 (HA mass fraction) (*P* = 0.730). Given the fluctuations in the trend for toughness between the consecutive HA concentration levels (*i.e*., no changes in the magnitude versus positive/negative changes), clearly this simple analysis suggests that it is more straightforward to optimise the stiffness and strength (as compared to toughness) of the chitosan composite with respect to crystallization temperature. 

**Figure 3:**
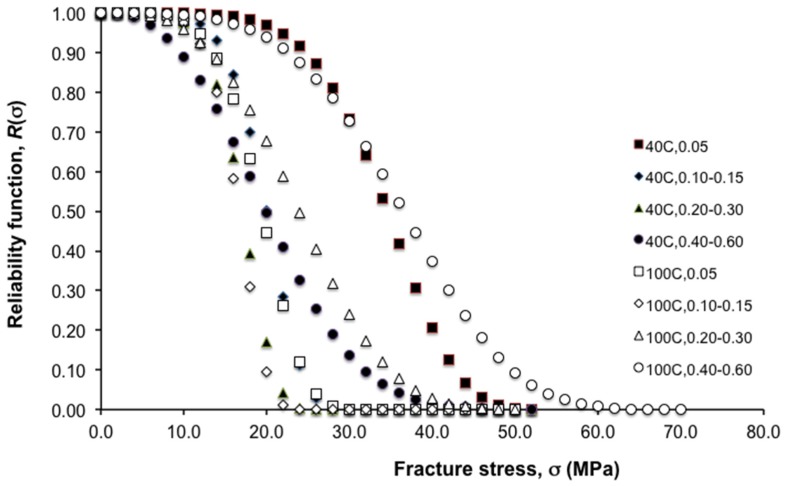
Plot of reliability function, *R*(σ), *versus* fracture stress in the fibre, σ, at varying hydroxyapatite (HA) concentrations and crystallization temperatures. Note: *R*(σ) = exp(−(σ/σ_0_)^β^), where σ_0_ and β are the characteristic strength and the Weibull modulus, respectively.

Table 2 shows the results of the analysis of the Weibull modulus, β, and the characteristic strength, σ_0_, of the chitosan fibre reinforced by HA nanoparticles. It is observed that β increases with increase in HA concentration, peaking at 0.10–0.15 (for both 40 °C and 100 °C levels). Thereafter, β decreases somewhat with an increase in HA concentration. With regards to the 40 °C crystallization temperature, σ_0_ is high at low HA concentration (*i.e*., 0.05); σ_0_ decreases when the HA concentration increases to 0.10–0.15 and fluctuates with further increase in HA concentration. With regard to the 100 °C crystallization temperature, while the σ_0_ is high at a low particle concentration (*i.e.*, 0.05), the magnitude decreases when the HA concentration increases to 0.10–0.15; thereafter, the σ_0_ increases dramatically with increasing HA concentration. Figure 3 illustrates how these parameters influence the reliability of the fibres using a plot of the reliability function, *R*(σ), *versus* fracture stress in the fibre, σ, for all the levels of HA concentration and crystallization temperature considered in this study. Thus the form (parameterized by β) of the curves corresponding to 40 °C (0.10–0.15, 0.20–0.30, 0.40–0.60 HA concentration) levels and 100 °C (0.05, 0.10–0.15, 0.20–0.30 HA concentration) levels features a very narrow spreads of strength variability as compared to 40 °C (0.05 HA concentration) levels and 100 °C (0.40–0.60 HA concentration) levels. Correspondingly, the σ_0_ for the 40 °C (0.05 HA concentration) and 100 °C (0.40–0.60 HA concentration) levels are dramatically larger than those associated with the other levels.

**Table 2:** Analysis of the Weibull modulus, β, and the characteristic strength, σ_0_, of chitosan fibres reinforced by hydroxyapatite (HA) nanoparticles.

Crystallization Temperature (°C)	HA Concentration (mass fraction)	β	σ_0_ (MPa)
40	0.05	5.6	36.9
40	0.10–0.15	6.3	21.2
40	0.20–0.30	6.1	18.2
40	0.40–0.60	2.6	23.0
100	0.05	5.3	20.8
100	0.10–0.15	6.6	17.6
100	0.20–0.30	3.2	26.8
100	0.40–0.60	3.9	40.1

## 3. Discussion

Our hypothesis that particle concentration affects the stiffness, strength and toughness holds true. In all cases, it is shown that the stiffness, strength and toughness are high at low HA concentrations but decrease as the HA concentration increases. There exists a (critical) HA concentration that leads to a minimum value for the mechanical properties such that any increase in HA concentration thereafter would result in an increase in the stiffness, strength and toughness; in all the cases, it is shown that the critical HA concentration appears at around 0.30–0.40. Below the critical HA concentration, the negative changes in the stiffness, strength and toughness with increasing HA concentration imply that HA particle agglomeration occurs; this agglomeration effect is consistent with many systems that comprise nanoparticle-reinforced composite material [21]. We speculate that the critical HA concentration corresponds to a state of saturation for the formation of particle agglomerates such that further increases in HA concentration would mean that particle dispersion is energetically more favourable than particle agglomeration. Thus, beyond the critical HA concentration, the positive changes in the stiffness, strength and toughness with increasing HA concentration suggest that the particle dispersion effects are predominate over particle agglomeration effects. Analysis of the reliability of these fibres reveals that the variability of the strength decreases (in other words β increases) with increase in HA concentration, peaking at 0.10–0.15 (for both 40 °C and 100 °C levels). Thereafter, the variability of the strength increases (*i.e*., β decreases) somewhat with an increase in HA concentration. 

**Figure 4:**
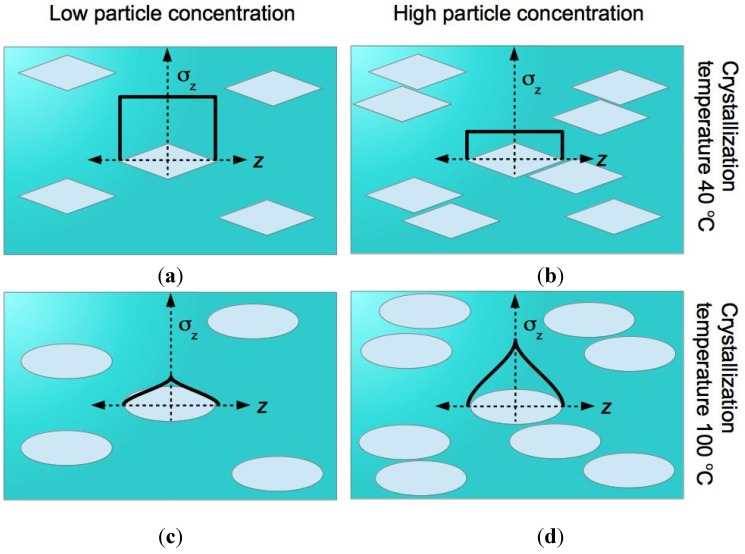
Schematic of HA-nanoparticle-reinforced chitosan composite fibre. (**a**,**c**) and (**b**,**d**) depict HA in the chitosan matrix at low and high concentrations, respectively; (**a**,**b**) and (**c**,**d**) depict HA particles corresponding to crystallization temperature at 40 and 100 °C, respectively. Overlaying one of the particles in each panel is a sketch of the graph of axial stress (σ*_z_*) in the particle versus distance (*z*) along the particle axis.

Our hypothesis that crystallization temperature affects the stiffness, strength and toughness holds true only at certain HA concentration levels. It has been reported that HA nanoparticles are slender with a characteristic straight taper at both ends when crystallised at 40 °C [7] (Figure 3). On the other hand, the morphology of the HA nanoparticles at 100 °C crystallization temperature appears as near-ellipsoid [7] (Figure 4). The effects of particle shape on the mechanics of particle reinforcement of composite materials have been reported elsewhere [22,23,24,25,26,27,28]. The findings from previous studies conclude that the magnitude of the stresses in a particle with straight tapered ends is more uniformly distributed than in an ellipsoidal particle [22,23,24,25,26,27,28] (Figure 4). In order to provide effective reinforcement, ideally the particle would have the capacity to take up high stresses (hence lowering the stresses in the surrounding matrix material), but stress concentrations, approaching the fracture stress, should be avoided [22,23,25,27]. In other words, the magnitude of the stress needs to be as high as possible while the peak stress as low as possible [22,23,25,27]. These arguments suggest that the HA particle crystallised at 40 °C are effective in taking up high stresses (as well as providing for uniform stress distribution) at low HA concentration (Figure 3). However, at high HA concentration, the capacity to take up high stresses may be compromised by particle agglomeration effects. When considering the case of low HA concentration, HA particles crystallised at 100 °C are not as effective as those crystallised at 40 °C with regards to taking up high stresses and uniformity of stress distribution. However, when considering the case of high HA concentration, for HA particle crystallised at 100 °C, the capacity to take up high stress would be predominate over the agglomeration effects (Figure 4); for HA particle crystallised at 40 °C, the agglomeration effects predominates over the capacity to take up high stress (Figure 4). 

The hypothesis that the effect of any one of the two factors on the stiffness, strength and toughness is dependent on the other factor hold true. Herein lies the importance of searching for interactions between the parameters. For investigations that involve either parameter as singly applied treatment, it is easy to see that the conclusion of the main effects could be misleading as the results could be masked by the effects of the other parameter. In our case, masking is most appreciable when we are concerned with the effects of crystallization temperature on the mechanical properties at a given HA concentration level. Altogether, it is important to note that the issues arising from our findings concerning the main effects and the effects arising from interaction have not been addressed in previous studies [5].

We note that an alternative to the two-factor ANOVA is the Taguchi method, which typically requires fewer combinations of the parameter levels, instead of the full factorial, for executing the experiments [17]. For these reasons, the Taguchi method has been applied to solve many industrial processes, as well as in research and development activities [29]. While the full-factorial approaches are generally complex because they require a large number of experiments when the number of factors increases, in the current context, it is feasible to apply the full-factorial approach because we are dealing with only two processing variables, namely particle concentration and crystallization temperature (with two and four levels, respectively), which are assumed to be industrially important parameters. Hence, we have chosen to model the experimental data using the two-factor ANOVA, which is a simpler statistical model with fewer simplifying assumptions, to investigate the sensitivity of the elasticity of the HA-reinforced chitosan composite fibres to changes in the two parameters; our model is the simplest which enables this question to be answered. It is worth mentioning that some levels assigned to the variables have never been previously investigated, e.g., the response of the material under varying crystallization temperature at a given high HA concentration level, *i.e.*, 0.40–0.60 (HA mass fraction); hence, this study is an attempt not only to optimize the operating conditions of the wet synthesis of HA but also to simultaneously synthesize the HA particles under relatively novel conditions. 

## 4. Experimental Methods

The synthesis of the chitosan composite fibres, followed by mechanical testing to derive the load-deformation curves obtained by Xie *et al.* [5] were the starting point for the re-analysis of data necessary for the design of the experiment presented below. In summary, the hydroxyapatite nanocrystals were prepared by adding 250 mL of 0.12M H_3_PO_4_ to 250 mL of 0.2M Ca(OH)_2_. HA nanocrystals were precipitated at 40 °C and 100 °C respectively; the reaction in the medium was terminated at pH = 7.4. The white precipitates were collected after sedimentation. The chitosan solution (10 mL, 3% w/v) was prepared by stirring chitosan in acetic acid (1% w/v). A suspension (5 mL), containing precipitates of HA, was mixed with the chitosan solution (10 mL). With the help of a coagulant (NaOH 10% w/v) solution a predrawn wet spun fiber extrusion process [30] was used to extrude fibres from the chitosan-HA mixture (at a rate of 3.1 cm/min). At the exit, the fibres were collected into a coagulant solution (NaOH 10% w/v) and subsequently removed from the coagulant solution. The fibres possessed diameters ranging 174.1 to 240.3 μm. The fibres were sectioned to lengths of 150 mm; each test specimen was stretched to rupture using a tensile machine (E1000, Instron, Norwood, MA, USA) at a displacement rate of 0.04 mm/s.

Starting with the original records of the stress-strain data of each sample, we quantified the stiffness, strength and toughness of the chitosan composite fibre. The strength, stiffness and toughness were determined from the maximum stress, the gradient at the point of inflection along the stress–strain curve of each sample and the area under the curve from the origin to the maximum stress, respectively.

In considering the two processing parameters, HA concentration and crystallization temperature, we assigned the former to four levels, *i.e*., 0.05, 0.10–0.15, 0.20–0.30, 0.40–0.60 (mass fraction of HA, relative to chitosan composite), and the latter to two levels, *i.e.*, 40 °C and 100 °C, in this optimization study. Note that some of the HA concentration levels were defined by a range of values for the purpose of ensuring an adequate number of specimens for the respective level. A total of eight different combinations of crystallization temperature (two levels) and HA concentration (four levels) was investigated. 

A check on the nature of the mechanical properties data showed that the deviations of the data from the regression line (residuals) follow, to a large extent, a normal distribution and uniform variance (no appreciable trend, such as fan shape). Since these satisfied the criteria for ANOVA, we then carried out two-factor ANOVA (by applying the design of experiment approach that considers the general linear model), together with the Tukey test for the comparison of the mean values (family error rate = 0.01), to analyze the sensitivity of the respective mechanical parameter to particle concentration and crystallization temperature. Minitab software (version 16) was used to carry out the statistical analysis. Differences due to the treatment were considered significant if the *P*-value < 0.05. For informational purposes, the plots for the main and interaction effects were displayed using the representative (mean) values of the mechanical parameters. Where main effects were masked (*P* > 0.05) by interaction effects (*P* < 0.05), the relevant levels were subjected to further analysis using the Student *t*-test (two sample) method, e.g., comparing the specimens between the 40 and 100 °C crystallization temperature levels at the respective HA concentrations.

Finally, we note that HA agglomerates, or even local non-uniform dispersion of HA particulates within the chitosan fibre, can result in defects which act as stress intensifiers. As the stress intensifies, eventually chemical bonds within the defects are broken, and this results in cracks in the fibre. Adapting the method for fracture analysis (probabilistic approach) from a previous study [31], we note that from Weibull’s empirical law, the cumulative distribution function (*C*) of the fracture stress of the chitosan composite fibre, σ, for determining failure due to flaws is given by *C*(σ) = 1 − exp(−[σ/σ_0_]^β^). Here, β and σ_0_ represent the Weibull modulus and the characteristic strength, respectively. In particular, β parameterizes the variability of σ; low β values correspond to high variability and vice versa. σ_0_ is the stress value at which 63% of the fibres have fractured. In strength definition, we have the reliability function *R*(σ) = exp(−{σ/σ_0_}^β^) which refers to the proportion of the population of specimens sampled which will have a survived at fracture stress σ. To evalue β and σ_0_ for the different treatment groups, we first determined the median rank position (MR, to order of magnitude, this is identified with *R*) for each experimentally derived value of σ. This was carried out by ranking the σ data in ascending magnitude. The corresponding estimates of MR was evaluated using the equation MR = {*i* − 0.3}{*n* + 0.4}^−1^, where *n* represents the size of the treatment group and *i* is the position of the corresponding σ. We then fitted straight lines to the Weibull plot of log(log(1/{1–MR(σ)})) versus log(σ) for each group. Here we note that the value of β was identified with the slope of the respective straight lines while the value of σ_0_ was found by equating −βlog(σ_0_) to the y-intercept of the straight line. 

## 5. Conclusions

We have investigated the main effects of HA concentration and crystallization temperature on the stiffness, strength and toughness of wet-spun HA-reinforced chitosan composite fibres by evaluating the experimental data of their respective mechanical properties using a two-factor ANOVA approach. We summarized our findings as follows:

- The mechanical properties of the chitosan composite fibre are sensitive to HA concentration and crystallization temperature;
- However, owing to interactions between the two factors, the changes in the mechanical properties due to varying HA concentration also depend on the crystallization temperature (and vice versa);
- There exists a critical HA concentration level at which the magnitude of the respective mechanical properties is a minimum.
